# Risk factors for disease-related deterioration following diagnostic bronchoalveolar lavage procedures in diffuse lung disease: a case-control study

**DOI:** 10.7717/peerj.9864

**Published:** 2020-09-04

**Authors:** Yuko Usagawa, Kosaku Komiya, Mari Yamasue, Kazuhiko Hashinaga, Eri Mizukami, Kenji Umeki, Shin-ichi Nureki, Masaru Ando, Kazufumi Hiramatsu, Jun-ichi Kadota

**Affiliations:** 1 Department of Respiratory Medicine and Infectious Diseases, Oita University, Yufu, Japan; 2 Department of Medical Safety Management, Oita University, Yufu, Japan

**Keywords:** Bronchoalveolar lavage, Acute exacerbation, Diffuse lung disease, Idiopathic pulmonary fibrosis, Eosinophilic pneumonia

## Abstract

**Background:**

Although the risk factors for diagnostic bronchoalveolar lavage (BAL)-induced acute exacerbations in patients with idiopathic pulmonary fibrosis (IPF) have been previously reported, no study has assessed these in patients with non-IPF. We aimed to identify the risk factors for BAL-induced disease deterioration (BAL-DD) in all types of diffuse lung disease.

**Methods:**

Patients with diffuse lung disease who underwent BAL at our hospital from April 2012 to November 2017 were retrospectively analyzed. The patient information, laboratory data, radiological findings, and BAL fluid analysis results in patients who developed BAL-DDs were compared with those in patients who did not.

**Results:**

BAL-DDs occurred in 14 (3.3%) of the 429 patients included the study. The BAL-DD group had a significantly poorer performance status, higher C-reactive protein level, lower partial pressure of oxygen in the arterial blood at rest, greater proportion of desaturation on exertion and cases having followed a progressive clinical course before BAL, and more extensive consolidation and ground-glass opacity on chest high-resolution computed tomography (HRCT) than the non-BAL-DD group. A high total cell concentration and an increased number of eosinophils in the BAL fluid were more frequently found in patients with BAL-DD than in those without.

**Conclusions:**

Patients with decreased physical activity level, increased level of inflammatory markers, low oxygenation status, and extensive lung involvements on chest HRCT and following a progressive clinical course before BAL may be warned of the BAL-DD risk. Elevated eosinophil counts in the BAL fluid could be associated with the triggering of BAL-DDs.

## Introduction

In the 1980s, bronchoalveolar lavage (BAL) was established as a method for diagnosing diffuse lung disease (Reynolds, 1987). It is widely accepted that this method obtains useful alveolar-level information about the disease state (Meyer et al., 2012). A BAL fluid analysis includes not only microorganism culturing but also an analysis of the cellular constituents or CD4/CD8 ratio of lymphocytes for the differential diagnosis of interstitial lung diseases, including idiopathic pulmonary fibrosis (IPF), interstitial pneumonia with connective tissue disease (CTD-ILD), sarcoidosis, or eosinophilic, drug-induced, or hypersensitivity pneumonia.

Although BAL is generally regarded as a safe diagnostic procedure (Klech & Hutter, 1990), BAL-induced acute exacerbations (BAL-AEs) of lung involvement, especially in IPF, which may lead to lethal conditions, have been increasingly recognized. The risk of a BAL-AE in patients with IPF has been estimated to be 1.99%–2.4% (Hiwatari et al., 1994; Sakamoto et al., 2012), and a lower forced vital capacity (FVC) and carbon monoxide diffusing capacity (DL_CO_), increased C-reactive protein (CRP) level, and high body temperature have been suggested as risk factors (Sakamoto et al., 2012). However, these studies only assessed BAL-AEs in patients with IPF. BAL is usually performed to diagnose unknown lung disease rather than typical IPF. In fact, the recent guidelines for IPF do not necessarily require BAL for the diagnosis (Raghu et al., 2018). Little is known about the features of BAL-induced disease exacerbations in patients with diffuse lung diseases other than IPF, and no published studies have assessed the risk factors in these patients. Thus, in the present study, we aimed to identify the risk factors for BAL-induced disease deteriorations (BAL-DDs) in all types of diffuse lung disease.

## Materials and Methods

### Patients

As a case–control study, we retrospectively included consecutive patients with diffuse lung disease who underwent a diagnostic BAL procedure in Oita University Hospital, Yufu, Japan, from April 2012 to November 2017, and then patients were divided into the case group who met the BAL-DDs criteria (defined later) or the control group who did not meet the criteria. Since no reports of BAL-DDs have been described in patients who only show lymph node enlargement, such as sarcoidosis without consolidation or ground-grass attenuation in the lung fields, we excluded these cases. Cases in which BAL was performed for treatment (e.g., pulmonary alveolar proteinosis) were also excluded.

The study protocol was approved by the institutional ethics committee (approval number, 1507; approval date, October 12, 2018). The need for informed consent was waived because of the retrospective nature of the study, and information on this study was posted at the hospital with a method to opt out on our web page (https://www.med.oita-u.ac.jp/naika2/for_patients/pdf/04.pdf).

### The procedure, processing and analysis of BAL

BAL and the analysis including cell counts were performed by professional physicians certified by the Japan Society for Respiratory Endoscopy in Oita University Hospital as described previously (Sakamoto et al., 2004). After local anesthesia with 4% lidocaine, the patient was premedicated intramuscularly with pethidine hydrochloride (17.5–35 mg). A flexible bronchoscope (BF-260, Olympus, Tokyo) was wedged into the selected bronchopulmonary segment, typically in the middle or lingular lobe for lavage. A 50-mL sterile physiological saline solution at body temperature was instilled through the bronchoscope, and the fluid was immediately retrieved by gentle suction at a reduced pump pressure. Saline instillation was performed two or three times, resulting in 100 or 150 ml in total. The collected BAL fluid was immediately processed, filtered through gauze, and centrifuged at 550 rpm for 5 min. The total cells were counted in a hemocytometer. The slides were stained with May-Grunwald-Giemsa stain, and 900 cells were counted for the cell differentials with microscope objective lens of 40 × or 100 ×.

### Outcomes and definitions

The term “BAL-AE” is rarely used in patients with non-IPF diffuse lung disease because “AE” was defined as deterioration in patients with IPF. It is difficult to clarify whether the worsening that occurred in AE was similar to that observed in IPF or whether it was a disease-related clinical deterioration in patients with non-IPF. We used the term BAL-DDs as the outcome in this study, defined according to the 2004 criteria for AE in IPF (Collard et al., 2007) and the new diagnostic criteria of International Working Group Report on AE of IPF, which were reported in 2016 (Collard et al., 2016). BAL-DD was defined as the case that met all of the following items: (1) worsening of dyspnea within one month after BAL; (2) emergence of new ground-glass opacities or consolidation within one month after BAL; (3) oxygenation deterioration with a decline of ≥10 mmHg in the partial pressure of oxygen in the arterial blood (PaO_2_) from the level right before BAL; and (4) no clinical evidence of congestive heart failure, pneumothorax, pleural effusion and pulmonary embolism as a cause of the acute worsening of the patient’s condition. The control case was defined as the case that did not meet the above-mentioned the BAL-DD criteria.

In this study, in addition to one-point patient’s characteristics right before BAL, the clinical course within one month before the BAL procedure was also documented because physicians often need to make difficult decisions whether BAL should be performed for a patient taking a progressive clinical course. We defined the progressive case before BAL as a patient who met all of the following items: (1) worsening of dyspnea within one month before BAL; (2) emergence of new ground-glass opacities or consolidation within one month before BAL; (3) oxygenation deterioration with a decline of ≥10 mmHg in the partial pressure of oxygen in the arterial blood (PaO_2_) from the previous level within one month; and (4) no clinical evidence of congestive heart failure, pneumothorax, pleural effusion, and pulmonary embolism as a cause of the acute worsening of the patient’s condition.

### Data collection

We collected the following patient information and clinical data from medical records within two weeks before BAL: sex, age, final diagnosis, physical activity level, body temperature, desaturation on exertion according to 6-min walking test or clinical notes, serum CRP levels, Krebs von den Lungen-6 (KL-6) as a marker for the activity of interstitial pneumonia, and PaO_2_, vital capacity (VC), %VC, FVC, %FVC, forced expiratory volume in one s (FEV_1_), FEV1% and percentage of the diffusing capacity of the lung carbon monoxide (DL_CO_) on a respiratory function test before the BAL procedure. The physical activity level was assessed according to the Eastern Cooperative Oncology Group performance status (PS) (Oken et al., 1982). The regular use of glucocorticoid, immunosuppressant and antifibrotic agent for underlying diseases before the BAL procedure was also assessed.

The high-resolution computed tomography (HRCT) findings before the BAL procedure were evaluated for signs of ground-glass opacity, consolidation, honeycombing, bronchiectasis, and emphysema. To differentiate infiltration and ground-glass opacity by the BAL procedure itself from those by DDs, newly developed lung involvements in segments other than the segment that normal saline was injected for BAL were defined as DD-consistent findings. Two respiratory physicians (YU and KK) independently reviewed the chest HRCT features. Any disagreement between the presence of the findings and HRCT diagnosis in each case was resolved by a review conducted by the same two physicians to reach a consensus. The extension of ground-glass opacity and consolidation was also evaluated.

The following information from the BAL procedure was also documented: collection rate, total cell concentration, and cellular constituents including the numbers of macrophages, lymphocytes, neutrophils and eosinophils. Furthermore, in patients with BAL-DDs, a detailed clinical information, including the timing of the BAL-DD, treatment regimen, and outcome, was collected to clarify their prognosis after the BAL-DD.

### Statistical analyses

The odds ratio of each variable for BAL-DD was analyzed using a logistic regression in two models including or excluding the progressive cases before BAL. The Mann–Whitney U-test was used to compare the eosinophil counts between independent two groups because of non-normal distribution resulted from small number of patients. Two-tailed analyses were performed, and *P* values < 0.05 were considered statistically significant. All statistical analyses were performed using the IBM SPSS statistics software program (version 22; IBM SPSS, Tokyo, Japan).

## Results

### Patients’ characteristics

**Figure 1:**
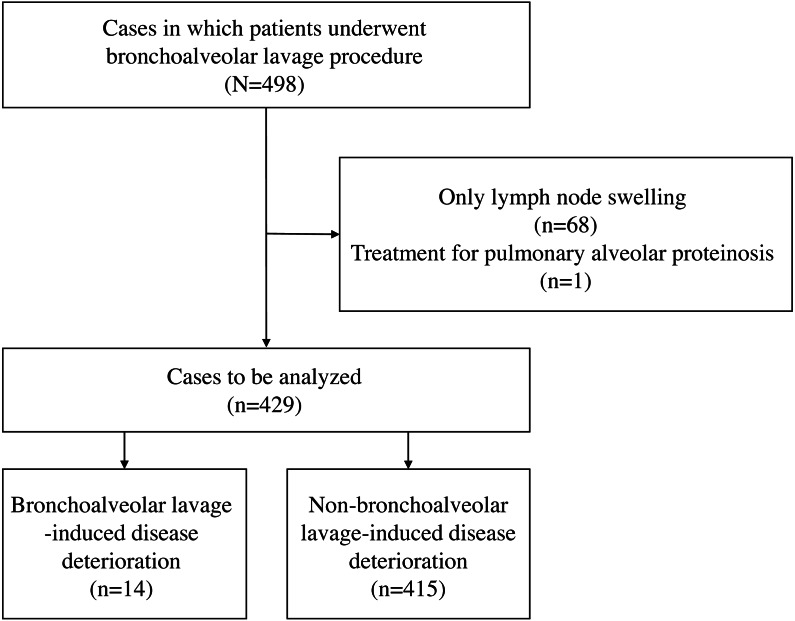
A flowchart of the participants evaluated over the course of the study and the number of patients in each group.

A total of 498 BAL procedures were performed in the study period, and we excluded 68 patients who had only lymph node enlargement and one who underwent BAL as treatment for pulmonary alveolar proteinosis. Thus, we finally included 429 patients: IPF in 12 and non-IPF in 417 patients. Fourteen (3.3%) of 429 patients developed BAL-DD (Fig. 1). When the patients who followed a progressive clinical course before BAL were excluded, the incident rate of BAL-DD was 2.1% (8/380). None of the patients with IPF experienced BAL-AE in this study.

In the analyses including the patients who followed a progressive clinical course before BAL, the BAL-DD group had a significantly poorer PS, higher body temperature and CRP level, lower PaO_2_, and a greater number of cases with desaturation on exertion and following a progressive clinical course before the BAL than the non-BAL-DD group (Table 1). No significant differences in the age, gender, KL-6 level, proportion of body temperature ≥ 38 °C, other respiratory functional parameters or regular use of glucocorticoid, immunosuppressant and antifibrotic agent for underlying diseases before BAL procedure were noted between these two groups. The extension of consolidation and ground-glass opacity was seen in the BAL-DD group significantly more frequently than in the non-BAL-DD group. Moreover, the incidence proportion of honeycombing, bronchiectasis, and emphysematous changes in the two groups did not differ significantly.

**Table 1:** Clinical features, laboratory findings, respiratory functional parameters and radiological findings before BAL procedures in patients with and without BAL-induced disease-related deterioration, including progressive cases before BAL.

	Non-BAL-DD (*n* = 415)	BAL-DD (*n* = 14)	Odds ratio (95% CI)	*p*
Age, years	67 (58–75)	68 (63–79)	1.004 (0.966–1.043)	0.849
Female	195 (47.0)	5 (35.7)	0.627 (0.207–1.902)	0.409
PS (%)			1.893 (1.180–3.038)	0.008
0	336 (81.0)	10 (71.4)		
1	19 (4.6)	0 (0)		
2	21 (5.1)	1 (7.1)		
3	8 (1.9)	3 (21.4)		
4	1 (0.2)	0 (0)		
unknown	30 (7.2)	0 (0)		
Body temperature (°C)	36.5 (36.2–36.7)	36.7 (36.3–37.5)	2.639 (1.205–5.781)	0.015
Body temperature ≥38 °C (%)	6 (1.4)	0 (0)	n.a.	0.999
CRP (mg/dL)	0.22 (0.08–1.06)	4.23 (1.96–10.09)	1.139 (1.059–1.225)	<0.001
KL-6 (U/ml)	589 (338–1,110)	460 (251–1,013)	1.000 (1.000–1.000)	0.896
KL-6 ≥500 U/ml (%)	224 (54.0)	6 (42.9)	0.689 (0.227–2.086)	0.510
PaO_2_ (Torr)	83.0 (72.0–92.0)	74.0 (59.5–83.5)	0.957 (0.921–0.994)	0.024
PaO_2_≤60 Torr (%)	35 (8.4)	4 (28.6)	4.825 (1.414–16.470)	0.012
Desaturation on exertion	151 (37.3)	11 (84.6)	9.252 (2.023–42.302)	0.004
VC (L)	2.53 (2.04–3.13)	2.49 (1.65–2.62)	0.535 (0.192–1.488)	0.231
%VC (%)	87.7 (74.1–101.4)	79.7 (67.6–84.8)	0.987 (0.943–1.014)	0.224
FVC (L)	2.52 (2.01–3.07)	2.40 (1.52–2.62)	0.511 (0.182–1.440)	0.204
%FVC (%)	87.2 (71.9–101.9)	69.5 (67.1–84.8)	0.973 (0.939–1.009)	0.137
FEV1.0 (L)	1.98 (1.59–2.43)	1.81 (1.31–2.17)	0.534 (0.151–1.881)	0.329
FEV1.0% (%)	80.1 (74.5–84.7)	82.8 (75.4–88.1)	1.019 (0.936–1.110)	0.663
%DL_CO_ (%)	79.8 (61.5–96.1)	52.6 (43.5–77.3)	0.943 (0.886–1.004)	0.067
Progressive case before BAL procedure	35 (8.4)	6 (42.9)	8.143 (2.674–24.800)	<0.001
Glucocorticoid	31 (7.5)	1 (7.1)	0.953 (0.121–7.526)	0.963
Immunosuppressant	6 (1.4)	0 (0)	n.a.	n.a.
Antifibrotic agent	1 (0.2)	0 (0)	n.a.	n.a.
Ground-glass opacity	370 (89.2)	13 (92.9)	1.581 (0.202–12.372)	0.663
Consolidation	122 (29.4)	9 (64.3)	4.323 (1.420–13.162)	0.010
Extension of GGO (number of lobes)	3 (2–4)	4 (3–5)	1.537 (1.018–2.320)	0.041
Extension of consolidation (number of lobes)	0 (0–1)	2 (0–2)	1.643 (1.180–2.288)	0.003
Honeycombing	60 (14.5)	2 (14.3)	0.986 (0.215–4.517)	0.986
Bronchiectasis	109 (26.3)	4 (28.6)	1.123 (0.345–3.654)	0.847
Emphysema	79 (19.0)	3 (21.4)	1.160 (0.316–4.256)	0.823

**Notes.**

Data are presented as the number (%) or median (interquartile range).

BAL bronchoalveolar lavage CI confidence interval CRP C-reactive protein DD disease-related deterioration DLCO carbon monoxide diffusing capacity FEV1.0 forced expiratory volume in one second FVC forced vital capacity GGO ground-glass opacity KL-6 Krebs von den Lungen-6 n.a. not available PaO_2_ partial pressure of oxygen in arterial blood PS perfoemances status VC vital capacity

When the patients who followed a progressive clinical course before BAL were excluded, the BAL-DD group had a significantly poorer PS, higher CRP level, and greater number of cases with desaturation on exertion than the non-BAL-DD group (Table 2). The extension of consolidation and ground-glass opacity was seen in the BAL-DD group significantly more frequently than in the non-BAL-DD group. Moreover, the incidence proportion of honeycombing, bronchiectasis, and emphysematous changes in the two groups did not differ significantly as well as the analysis including the patients who followed a progressive clinical course before BAL.

**Table 2:** Clinical features, laboratory findings, respiratory functional parameters and radiological findings before BAL procedures in patients with and without BAL-induced disease-related deterioration, excluding progressive cases before BAL.

	Non-BAL-DD (*n* = 380)	BAL-DD (*n* = 8)	Odds ratio (95% CI)	*p*
Age, years	67 (58–75)	68 (63–79)	1.000 (0.951–1.050)	0.990
Female	185 (48.7)	2 (25.0)	0.351 (0.070–1.763)	0.204
PS (%)			2.323 (1.111–4.860)	0.025
0	324 (85.3)	6 (75.0)		
1	16 (4.2)	0 (0)		
2	16 (4.2)	1 (12.5)		
3	2 (0.5)	1 (12.5)		
4	0 (0)	0 (0)		
unknown	31 (8.2)	0 (0)		
Body temperature (°C)	36.4 (36.1–36.7)	36.6 (36.1–36.9)	2.883 (0.627–13.252)	0.174
Body temperature ≥38 °C (%)	2 (0.5)	0 (0)	n.a.	1.000
CRP (mg/dL)	0.20 (0.08–0.66)	3.19 (1.88–8.23)	1.192 (1.052–1.352)	0.006
KL-6 (U/ml)	587 (345–1,080)	307 (211–1,029)	0.999 (0.997–1.001)	0.325
KL-6 ≥500 U/ml (%)	205 (53.9)	2 (25.0)	0.320 (0.061–1.671)	0.177
PaO_2_ (Torr)	84 (75–93)	83 (74–95)	0.995 (0.941–1.053)	0.872
PaO_2_≤60 Torr (%)	12 (3.2)	0 (0)	n.a.	0.999
Desaturation on exertion	126 (33.2)	6 (75.0)	6.024 (1.199–30.272)	0.029
VC (L)	2.52 (2.03–3.13)	2.62 (1.85–2.71)	0.695 (0.193–2.498)	0.577
%VC (%)	88.3 (74.3–101.5)	76.4 (69.0–83.5)	0.973 (0.928–1.022)	0.274
FVC (L)	2.52 (2.00–3.08)	2.61 (1.79–2.69)	0.703 (0.196–2.516)	0.588
%FVC (%)	87.9 (72.2–102.1)	74.1 (67.7–83.3)	0.972 (0.927–1.020)	0.248
FEV1.0 (L)	1.99 (1.59–2.42)	2.17 (1.53–2.37)	0.973 (0.218–4.353)	0.972
FEV1.0% (%)	80.1 (74.4–84.6)	84.5 (80.8–92.2)	1.102 (0.971–1.250)	0.134
%DL_CO_ (%)	80.7 (62.4–96.3)	49.3 (41.5–66.9)	0.945 (0.882–1.013)	0.113
Progressive case before BAL procedure	n.a.	n.a.	n.a.	n.a.
Glucocorticoid	25 (6.6)	1 (12.5)	2.029 (0.240–17.142)	0.516
Immunosuppressant	6 (1.6)	0 (0)	n.a.	0.999
Antifibrotic agent	0 (0)	0 (0)	n.a.	n.a.
Ground-glass opacity	337 (88.7)	8 (100)	n.a.	0.998
Consolidation	107 (28.2)	5 (62.5)	4.252 (0.999–18.105)	0.050
Extension of GGO (number of lobes)	3 (2–4)	4 (3–5)	2.086 (1.098–3.961)	0.025
Extension of consolidation (number of lobes)	0 (0–1)	2 (0–2)	1.517 (0.953–2.416)	0.079
Honeycombing	58 (15.3)	1 (12.5)	0.793 (0.096–6.567)	0.830
Bronchiectasis	102 (26.8)	2 (25.0)	0.908 (0.180–4.574)	0.907
Emphysema	70 (18.4)	2 (25.0)	1.476 (0.292–7.468)	0.638

**Notes.**

Data are presented as the number (%) or median (interquartile range).

BAL bronchoalveolar lavage CI confidence interval CRP C-reactive protein DD disease-related deterioration DLCO carbon monoxide diffusing capacity FEV1.0 forced expiratory volume in one second FVC forced vital capacity GGO ground-glass opacity KL-6 Krebs von den Lungen-6 n.a. not available PaO_2_ partial pressure of oxygen in arterial blood PS perfoemances status VC vital capacity

### BAL fluid analyses

The collection rates in the BAL-DD and non-BAL-DD groups were quite similar, as shown in Table 3. The total cell concentration and number of eosinophils were significantly higher in the BAL-DD group than in the non-BAL-DD group. However, these significances were lost when the patients who followed a progressive clinical course before BAL were excluded.

**Table 3:** The BAL analysis results in patients with and without BAL-induced disease-related deterioration.

	Non-BAL-DD including progressive cases before BAL (*n* = 415)	BAL-DD including progressive cases before BAL (*n* = 14)	Odds ratio	*p*	Non-BAL-DD excluding progressive cases before BAL (*n* = 380)	BAL-DD excluding progressive cases before BAL (*n* = 8)	Odds ratio	*p*
Collection rate (%)	44.0 (32.0–55.3)	42.0 (32.7–48.3)	0.992 (0.958–1.028)	0.675	44.7 (32.0–55.3)	37.7 (30.8–46.5)	0.978 (0.934–1.024)	0.350
Total cell concentration (10^5^/ml)	2.62 (1.59–4.35)	4.53 (2.53–8.22)	1.155 (1.031–1.294)	0.013	2.46 (1.55–4.16)	4.37 (3.01–6.31)	1.115 (0.941–1.322)	0.209
Macrophage count (10^5^/ml)	1.62 (1.08–2.38)	1.88 (1.44–4.03)	1.349 (0.998–1.823)	0.052	1.63 (1.10–2.38)	2.49 (1.81–4.62)	1.499 (1.068–2.106)	0.019
Lymphocyte count (10^5^/ml)	0.31 (0.12–1.16)	0.44 (0.26–2.72)	1.098 (0.904–1.333)	0.346	0.29 (0.11–1.05)	0.41 (0.17–1.94)	0.956 (0.618–1.479)	0.841
Neutrophil count (10^5^/ml)	0.05 (0.02–0.17)	0.15 (0.06–0.48)	1.018 (0.721–1.437)	0.919	0.04 (0.02–0.13)	0.12 (0.04–0.41)	1.023 (0.651–1.608)	0.923
Eosinophil count (10^5^/ml)	0.02 (0.00–0.09)	0.24 (0.13–1.48)	1.536 (1.171–2.016)	0.002	0.02 (0.00–0.08)	0.18 (0.06–1.16)	1.262 (0.802–1.986)	0.314

**Notes.**

Data are presented as median (interquartile range).

BAL bronchoalveolar lavage DD disease-related deterioration

### The final diagnosis of diffuse lung disease

BAL was most frequently performed in patients diagnosed with unclassified interstitial pneumonia, followed by those diagnosed with sarcoidosis, CTD-ILDs, and organizing pneumonia (Table 4). Patients with eosinophilic pneumonia (4/15, 26.7%), CTD-ILDs (4/64, 6.3%), drug-induced interstitial pneumonia (1/17, 5.9%), infection (1/21, 4.8%), hypersensitivity pneumonia (1/23, 4.3%), and unclassified interstitial pneumonia (1/107, 0.9%) suffered from BAL-DDs. On the other hand, BAL-DDs did not occur in patients with IPF, organizing pneumonia, sarcoidosis, or lymphoproliferative disease. When the progressive cases before BAL were excluded, patients with eosinophilic pneumonia (2/10, 20.0%), infection (1/17, 5.9%), CTD-ILDs (2/58, 3.4%), and unclassified interstitial pneumonia (1/103, 1.0%) suffered from BAL-DDs. The diagnosis of eosinophilic pneumonia was significantly associated with BAL-DDs whether the progressive cases before BAL were included or excluded, as shown in Table 4.

**Table 4:** The diagnoses of patients with and without BAL-induced disease-related deterioration.

	Non-BAL-DD including progressive cases before BAL (*n* = 415)	BAL-DD including progressive cases before BAL (*n* = 14)	Odds ratio	*p*	Non-BAL-DD excluding progressive cases before BAL (*n* = 380)	BAL-DD excluding progressive cases before BAL (*n* = 8)	Odds ratio	*p*
Idiopathic pulmonary fibrosis	12 (2.9)	0 (0)	n.a.	0.999	12 (3.2)	0 (0)	n.a.	0.999
Interstitial pneumonia with connective tissue diseases	60 (14.5)	4 (28.6)	2.367 (0.719–7.790)	0.156	56 (14.7)	2 (25.0)	1.929 (0.380–9.797)	0.428
Organizing pneumonia	43 (10.4)	0 (0)	n.a.	0.998	38 (10.0)	0 (0)	n.a.	0.998
Drug-induced interstitial pneumonia	16 (3.9)	1 (7.1)	1.918 (0.236–15.578)	0.542	10 (2.6)	0 (0)	n.a.	0.999
Eosinophilic pneumonia	11 (2.7)	4 (28.6)	14.691 (3.983–54.180)	<0.001	8 (2.1)	2 (25.0)	15.500 (2.702–88.916)	0.002
Hypersensitivity pneumonia	22 (5.3)	1 (7.1)	1.374 (0.172–10.986)	0.764	19 (5.0)	0 (0)	n.a.	0.998
Unclassified interstitial pneumonia	106 (25.5)	1 (7.1)	0.224 (0.029–1.735)	0.152	102 (26.8)	1 (12.5)	0.389 (0.047–3.204)	0.380
Sarcoidosis	69 (16.6)	0 (0)	n.a.	0.997	69 (18.2)	0 (0)	n.a.	0.997
Infection	20 (4.8)	1 (7.1)	1.519 (0.189–12.197)	0.694	16 (4.2)	1 (12.5)	3.250 (0.377–28.020)	0.284
Lymphoproliferative disease	12 (2.9)	0 (0)	n.a.	0.999	10 (2.6)	0 (0)	n.a.	0.999
Others	44 (10.6)	2 (14.3)	1.405 (0.305–6.485)	0.663	40 (10.5)	2 (25.0)	2.833 (0.553–14.512)	0.211

**Notes.**

Data are presented as the number (%).

BAL bronchoalveolar lavage DD disease-related deterioration n.a. not available

Among 15 patients with eosinophilic pneumonia, 3 and 12 were diagnosed with acute and chronic eosinophilic pneumonia, respectively. The eosinophil counts in these two types of eosinophilic pneumonia were not statistically different (median, 4.22 vs. 1.45 × 10^5^ cells/mL, *p* = 0.225). Furthermore, no significant difference in the eosinophil counts was noted between the patients with eosinophilic pneumonia who developed DD (*n* = 4) and those who did not (*n* = 11) (median, 2.01 vs. 1.52 × 10^5^ cells/mL, *p* = 0.839).

**Table 5:** The clinical characteristics of the 14 patients with BAL-induced disease-related deterioration.

Case #	Age	Before BAL						TCC (10^5^/ml)	AM (%)	Ly (%)	Ne (%)	Eo (%)	BAL to AE (hours or days)	After BAL						Diagnosis
		BT (°C)	PaO_2_ (Torr)	CRP (mg/dL)	KL-6 (U/ml)	GGO on CT (lobe)	Consolidation on CT (lobe)							BT (°C)	PaO_2_(Torr), (O_2_ flow, L/min)	CRP (mg/dL)	KL-6 (U/ml)	GGO on CT (lobe)	Consolidation on CT (lobe)	
1	10s	36.6	71	1.9	162	5	2	4.28	51	13	2	34	7 d	37.6	69 (5)	11.6	182	n.a.	n.a.	CEP
2	80s	36.4	60	4.2	7,298	5	0	2.19	79	8	9	4	2 d	38.7	67 (10)	10.4	n.a.	5	4	CHP
3	60s	n.a.	n.a.	n.a.	n.a.	4	3	2.00	71	22	1	7	4 h	n.a.	n.a.	9.3	n.a.	5	2	ABPA
4	60s	36.9	80	7.3	998	3	2	12.42	40	42	13	5	5 d	36.9	82 (2)	3.5	n.a.	5	3	ANCA-IP
5	40s	37.7	50	15.6	460	3	3	12.63	12	51	3	35	2 h	39.4	114 (10)	14.4	n.a.	n.a.	n.a.	AEP
6	70s	37.6	50	17.4	180	0	5	2.35	37	19	20	25	2 h	36.7	70 (10)	23.3	n.a.	n.a.	n.a.	CEP
7	80s	36.9	83	3.2	292	4	2	6.61	26	40	31	3	8 h	38.5	63 (1)	11.5	n.a.	4	3	Hemoptysis
8	60s	36.1	59	1.5	805	4	2	4.61	32	63	1	5	5 d	38.1	76 (2)	9.2	757	n.a.	n.a.	Overlap syndrome
9	80s	37.5	74	12.0	387	4	0	8.09	64	30	6	0	2 h	39.1	64 (10)	12.6	n.a.	n.a.	n.a.	Unclassified IP
10	60s	36.5	84	1.7	1,436	5	0	5.42	91	2	2	5	20 d	38.0	56 (2)	6.0	1,377	5	4	SLE IP
11	60s	36.1	95	3.5	307	3	2	4.45	63	1	1	36	6 d	36.0	80 (1)	2.8	n.a.	n.a.	n.a.	CEP
12	60s	36.7	97	8.2	211	2	1	2.59	79	11	5	6	7 d	36.7	48 (RA)	12.8	n.a.	n.a.	n.a.	Infection
13	70s	36.1	81	2.0	1,029	5	0	4.27	88	9	3	0	10 d	38.1	71	9.5	640	n.a.	n.a.	ANCA-IP
14	70s	37.5	62	5.0	693	4	0	8.60	15	5	2	78	6 d	38.4	55 (4)	11.9	n.a.	5	1	Drug induced

**Notes.**

ABPA allergic bronchopulmonary aspergillosis AEP acute eosinophilic pneumonia AM alveolar macrophage ANCA-IP antineutrophil cytoplasmic antibody positive interstitial pneumonia BAL bronchoalveolar lavage BT body temperature CEP chronic eosinophilic pneumonia CHP chronic hypersensitivity pneumonia CRP C-reactive protein Eo eosinophils GGO ground-glass opacity IP interstitial pneumonia KL-6 Krebs von den Lungen-6 Ly lymphocytes n.a. not available Ne neutrophils RA room air TCC total cell count

Case #2, #4, #5, #6, #8 and #14 had progressive condition before BAL. Case #4 and #9 had honeycombing on chest CT before BAL. Case #2 and #4 died due to progressive respiratory failure.

### Details of the patients with BAL-DDs

**Figure 2:**
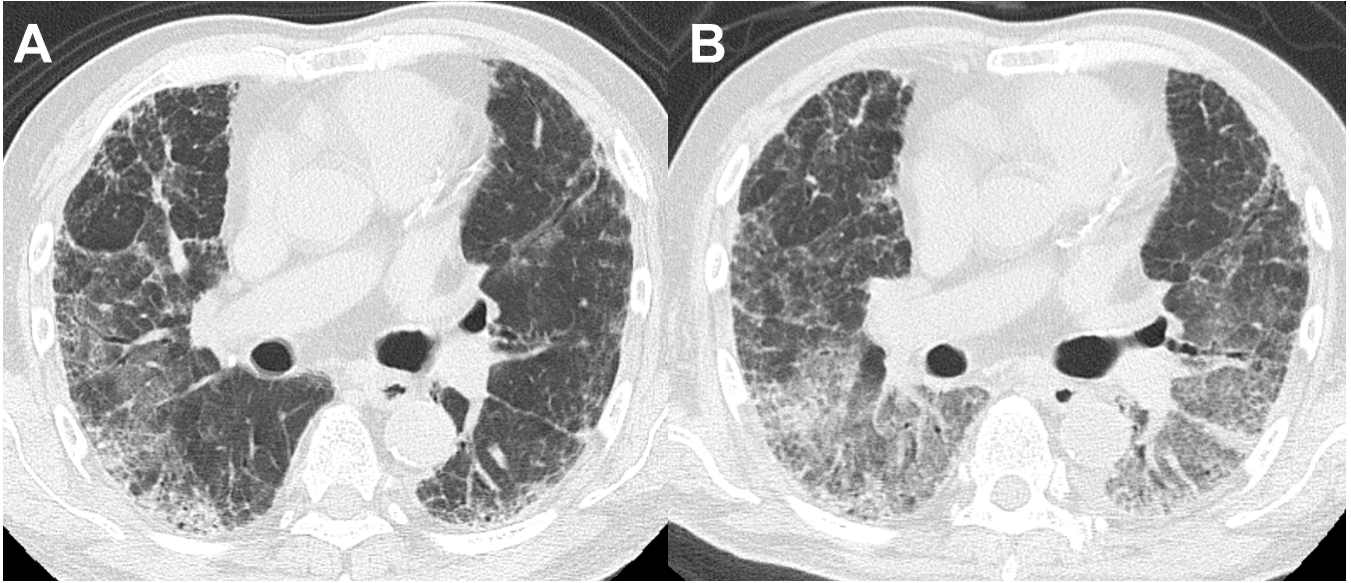
A patient in their 60s with chronic hypersensitivity pneumonia. (A) HRCT before the BAL procedure showed reticular shadows with bronchodilation in the peripheral lesion. (B) Two days after the BAL procedure, which was performed from B3b on the right lung, ground-glass opacities extended to both lungs.

**Figure 3:**
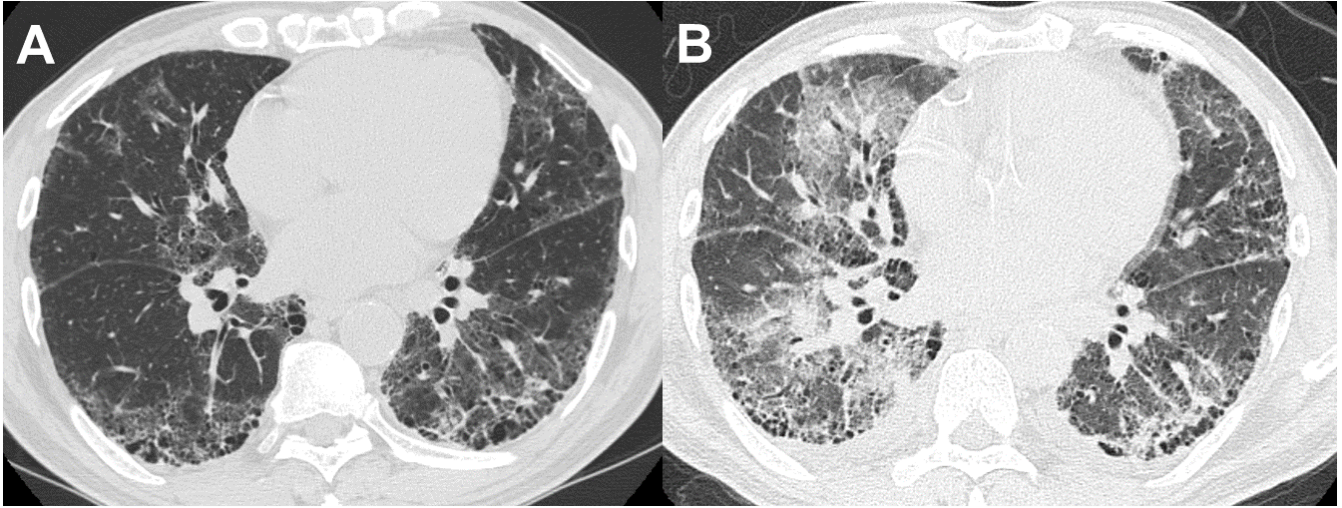
A patient in their 60s with interstitial pneumonia with connective tissue disease. (A) HRCT before the BAL procedure showed small cysts and reticular shadows. (B) HRCT at 20 days after the BAL procedure showed extensive ground-glass opacities in other areas.

Thirteen of the 14 patients with BAL-DDs experienced DDs within 10 days after the BAL procedure (Table 5). All patients with BAL-DDs received intensive treatment with high-dose systemic corticosteroids and broad-spectrum antibiotics. Consequently, 12 patients improved and were discharged from the hospital; however, two died due to progressive respiratory failure. The diagnoses of the two fatal cases were chronic hypersensitivity pneumonia and infection. In the former case, HRCT showed a reticular shadow in both lungs, and the serum was positive for antibody to *Trichosporon asahi*, which causes chronic summer-type hypersensitive pneumonia (Huang et al., 2006). After CTD-ILDs and drug-induced pneumonia were ruled out, the patient was diagnosed with chronic hypersensitivity pneumonia based on compatible radiological features. Two days after the BAL procedure, the oxygenation status had deteriorated, and the chest HRCT features had worsened (Figs. 2A and 2B). Despite intensive treatments with systemic glucocorticoids and antibiotics, the patients died of respiratory failure one month after the BAL procedure. The latter case showed extensive consolidation in the left lower lobe on HRCT and was admitted. Since antibiotic administration was not effective, we performed the BAL procedure at the superior lingular segment. The patient’s respiratory condition rapidly worsened seven days after the procedure. Despite the administration of systemic glucocorticoids with wide-spectrum antibiotics under mechanical ventilation, the patient died of respiratory failure three weeks after the BAL procedure. The autopsy revealed multiple pathological features, including bronchopneumonia, organizing pneumonia, diffuse alveolar damage, pulmonary hemorrhaging, and edema. No significant differences in the clinical signs, laboratory data, or HRCT features before and after BAL were noted between the patients who died and those who survived in the BAL-DD cases. However, chronic hypersensitivity pneumonia and infection with evidence of *Haemophilus influenzae* and *Streptococcus pneumoniae* isolation from sputum as the final diagnosis were only seen in the patients who died (Figs. 3A and 3B).

## Discussion

This study demonstrated that a poor PS, high serum CRP level, low oxygenation status, desaturation on exertion, extension of consolidation and ground-glass opacity on chest HRCT, and following a progressive clinical course within one month before BAL were associated with BAL-DDs. Elevated total cell concentration and eosinophil counts in the BAL fluid influenced the development of BAL-DDs. In fact, the diagnosis of eosinophilic pneumonia was significantly associated with BAL-DDs, whether or not patients who followed a progressive clinical course before BAL were included in the analysis. On the other hand, patients with IPF, organizing pneumonia, sarcoidosis, and lymphoproliferative diseases did not develop BAL-DDs. The frequency of BAL-DDs (3.3%) in our study, which included patients with any type of diffuse lung disease, seems to be slightly higher than that described in IPF, but the rate excluding progressive cases before BAL was identical with that in IPF.

We found that the BAL-DD group more frequently showed higher inflammatory marker levels and lower oxygenation status than the non-BAL-DD group, which was consistent with the findings of previous reports targeting patients with IPF (Sakamoto et al., 2012). No study has assessed the relationship between HRCT findings and BAL-AEs even in IPF. The extension of ground-glass opacity or consolidation can be correlated with the disease activity in acute or subacute noninfectious lung disease (Johkoh et al., 2000), so it is reasonable to obtain the results that the extension of consolidation or ground-glass opacity was associated with BAL-DDs.

Our results were also consistent with those of a previous study that reported that higher eosinophil (≥3.21%) percentages in the BAL fluid from patients with IPF were associated with a poor AE-free probability (Kakugawa et al., 2016). The mechanism underlying the occurrence of DD after BAL is still unclear, but an active inflammation status may be a crucial trigger. In addition to active inflammation, the spread of infection caused by the BAL procedure, atelectasis, and pulmonary injury induced by the washout of pulmonary surfactant (Lachmann, Robertson & Vogel, 1980; Russ et al., 2016) and the elevation of inflammatory cytokines, such as IL-6 and TNF *α*, in the blood and BAL fluid (Krause et al., 1997; Terashima et al., 2001) may be involved in the occurrence of BAL-DDs. The BAL-DD group in our study included four patients (26.7%) who had been diagnosed with eosinophilic pneumonia, which was consistent with the finding that the eosinophil percentage in the BAL fluid of the BAL-DD group was significantly higher than that of the non-BAL-DD group. BAL procedures may spread and stimulate eosinophils, and the activation of eosinophils can subsequently damage lung tissue via the release of cytokines, such as eosinophil cationic protein (Hallgren et al., 1987), major basic protein, and oxygen radicals, as well as the generation of peroxidase by halide-related oxidants (Henderson, Chi & Klebanoff, 1980). Among patients diagnosed with eosinophilic pneumonia, a higher eosinophil count in the BAL fluid may be related to the risk of BAL-DD despite of no statistical significance in this study probably due to the small number of patients with eosinophilic pneumonia.

While some studies have reported on BAL-AEs in IPF patients (Hiwatari et al., 1994; Sakamoto et al., 2012), none of the IPF patients experienced a BAL-AE in this study, probably due to the fact that the number of patients with IPF included in this study was relatively small. Since IPF can be diagnosed based on typical findings on chest HRCT and these cases do not necessarily require BAL (Raghu et al., 2018), the frequency of BAL procedure in patients with IPF was low. There is a possibility that some patients diagnosed with unclassified interstitial pneumonia (107 of 429) could have been diagnosed with IPF after further examinations such as video-assisted thoracic surgery. However, only the one case with unclassified interstitial pneumonia experienced BAL-DD, as shown in Table 3. At least, our results did not demonstrate that the diagnosis of IPF can be a risk factor for BAL-DD. Regarding the classifications of diffuse lung disease, the BAL-DD group predominantly included patients with CTD-ILD. No reports of BAL-DDs in patients with CTD-ILD have been described. Whether or not patients with CTD-ILD should undergo a diagnostic BAL procedure is controversial, as it does not always provide useful information for decision-making on treatment strategies or predicting the prognosis (Goh et al., 2007; Kowal-Bielecka, Kowal & Chyczewska, 2010; Volpinari et al., 2011). The indications for a diagnostic BAL procedure need to be carefully chosen when excluding infection or hemorrhaging.

The present study has several limitations. First, the study population included several patients with unclassified interstitial pneumonia. Thus, we cannot state which specific diffuse lung diseases except for eosinophilic pneumonia were associated with a greater risk of experiencing BAL-DD. Second, DD can be difficult to distinguish due to the natural disease process itself and the BAL procedure. We assessed whether or not patients met the criteria for following a progressive clinical course within one month before the BAL procedure. In the BAL-DD group, 6 (42.9%) of 14 patients met the criteria before the BAL procedure, and we cannot deny that these patients may have met the BAL-DD criteria as a result of the natural disease course. However, it is noted that 35 (85.3%) of the 41 patients who met the criteria for the progressive clinical course within one month before BAL did not develop BAL-DDs. Patients who followed a progressive clinical course before BAL may not necessarily develop BAL-DDs. Third, we could not obtain the clinically clear data or inflammatory markers immediately after BAL because most of the patients did not develop DDs and they are not required to have these evaluations as well as before BAL in the retrospective nature of this study. One study found that acute phase responses were detected at 24 h after BAL (Huang et al., 2006). If these post-BAL data could had been collected, some inflammatory markers may have been raised as possible predictive factors for BAL-DDs. Finally, the number of patients with BAL-DDs in this study was too small to conduct a multivariate analysis. Whether or not any of the potential risk factors for BAL-DD that were identified in the present study had confounding effects remains controversial.

## Conclusions

DD occurred after BAL procedures in some patients with non-IPF diffuse lung disease, especially those with eosinophilic pneumonia as a final diagnosis. The inflammatory marker levels, oxygenation status, chest HRCT findings, progressive clinical course within one month before BAL, and elevated eosinophil counts in the BAL fluid appear to be associated with BAL-DDs in patients with any type of diffuse lung disease. While the BAL procedure is a safe examination overall, physicians should be aware that even non-IPF diffuse lung disease may develop BAL-DDs, and patients with the aforementioned risk factors require careful follow-up after a BAL procedure. A large-scale study is required to verify these results after adjusting for other variables.

##  Supplemental Information

10.7717/peerj.9864/supp-1 Supplemental Information 1 Raw data

## Additional Information and Declarations

### Data Availability

The following information was supplied regarding data availability: The raw data is available as a Supplementary File.

## References

[ref-1] Collard HR, Moore BB, Flaherty KR, Brown KK, Kaner RJ, King Jr TE, Lasky JA, Loyd JE, Noth I, Olman MA, Raghu G, Roman J, Ryu JH, Zisman DA, Hunninghake GW, Colby TV, Egan JJ, Hansell DM, Johkoh T, Kaminski N, Kim DS, Kondoh Y, Lynch DA, Muller-Quernheim J, Myers JL, Nicholson AG, Selman M, Toews GB, Wells AU, Martinez FJ (2007). Acute exacerbations of idiopathic pulmonary fibrosis. American Journal of Respiratory and Critical Care Medicine.

[ref-2] Collard HR, Ryerson CJ, Corte TJ, Jenkins G, Kondoh Y, Lederer DJ, Lee JS, Maher TM, Wells AU, Antoniou KM, Behr J, Brown KK, Cottin V, Flaherty KR, Fukuoka J, Hansell DM, Johkoh T, Kaminski N, Kim DS, Kolb M, Lynch DA, Myers JL, Raghu G, Richeldi L, Taniguchi H, Martinez FJ (2016). Acute exacerbation of idiopathic pulmonary fibrosis. An international working group report. American Journal of Respiratory and Critical Care Medicine.

[ref-3] Goh NS, Veeraraghavan S, Desai SR, Cramer D, Hansell DM, Denton CP, Black CM, Du Bois RM, Wells AU (2007). Bronchoalveolar lavage cellular profiles in patients with systemic sclerosis-associated interstitial lung disease are not predictive of disease progression. Arthtitis and Rheumatism.

[ref-4] Hallgren R, Samuelsson T, Venge P, Modig J (1987). Eosinophil activation in the lung is related to lung damage in adult respiratory distress syndrome. American Review of Respiratory Disease.

[ref-5] Henderson WR, Chi EY, Klebanoff SJ (1980). Eosinophil peroxidase-induced mast cell secretion. Journal of Experimetnal Medicine.

[ref-6] Hiwatari N, Shimura S, Takishima T, Shirato K (1994). Bronchoalveolar lavage as a possible cause of acute exacerbation in idiopathic pulmonary fibrosis patients. Tohoku Journal of Experimental Medicine.

[ref-7] Huang YC, Bassett MA, Levin D, Montilla T, Ghio AJ (2006). Acute phase reaction in healthy volunteers after bronchoscopy with lavage. Chest.

[ref-8] Johkoh T, Tomiyama N, Honda O, Mihara N, Kozuka T, Maeda M, Hamada S, Naito H, Nakamura H, Ichikado K (2000). Acute and subacute non-infectious lung diseases: usefulness of HRCT for evaluation of activity especially in follow-up. Radiation Medicine.

[ref-9] Kakugawa T, Sakamoto N, Sato S, Yura H, Harada T, Nakashima S, Hara A, Oda K, Ishimoto H, Yatera K, Ishimatsu Y, Obase Y, Kohno S, Mukae H (2016). Risk factors for an acute exacerbation of idiopathic pulmonary fibrosis. Respiratory Research.

[ref-10] Klech H, Hutter C (1990). Side-effects and safety of BAL. European Respiratory Journal.

[ref-11] Kowal-Bielecka O, Kowal K, Chyczewska E (2010). Utility of bronchoalveolar lavage in evaluation of patients with connective tissue diseases. Clinics in Chest Medicine.

[ref-12] Krause A, Hohberg B, Heine F, John M, Burmester GR, Witt C (1997). Cytokines derived from alveolar macrophages induce fever after bronchoscopy and bronchoalveolar lavage. American Journal of Respiratory and Critical Care Medicine.

[ref-13] Lachmann B, Robertson B, Vogel J (1980). In vivo lung lavage as an experimental model of the respiratory distress syndrome. Acta Anaesthesiol Scand.

[ref-14] Meyer KC, Raghu G, Baughman RP, Brown KK, Costabel U, Du Bois RM, Drent M, Haslam PL, Kim DS, Nagai S, Rottoli P, Saltini C, Selman M, Strange C, Wood B (2012). An official American Thoracic Society clinical practice guideline: the clinical utility of bronchoalveolar lavage cellular analysis in interstitial lung disease. American Journal of Respiratory and Critical Care Medicine.

[ref-15] Oken MM, Creech RH, Tormey DC, Horton J, Davis TE, McFadden ET, Carbone PP (1982). Toxicity and response criteria of the Eastern Cooperative Oncology Group. American Journal of Clinical Oncology.

[ref-16] Raghu G, Remy-Jardin M, Myers JL, Richeldi L, Ryerson CJ, Lederer DJ, Behr J, Cottin V, Danoff SK, Morell F, Flaherty KR, Wells A, Martinez FJ, Azuma A, Bice TJ, Bouros D, Brown KK, Collard HR, Duggal A, Galvin L, Inoue Y, Jenkins RG, Johkoh T, Kazerooni EA, Kitaichi M, Knight SL, Mansour G, Nicholson AG, Pipavath SNJ, Buendia-Roldan I, Selman M, Travis WD, Walsh S, Wilson KC (2018). Diagnosis of idiopathic pulmonary fibrosis. An official ATS/ERS/JRS/ALAT clinical practice guideline. American Journal of Respiratory and Critical Care Medicine.

[ref-17] Reynolds HY (1987). Bronchoalveolar lavage. American Review of Respiratory Disease.

[ref-18] Russ M, Kronfeldt S, Boemke W, Busch T, Francis RC, Pickerodt PA (2016). Lavage-induced surfactant depletion in pigs as a model of the acute respiratory distress syndrome (ARDS). Journal of Visualized Experiments.

[ref-19] Sakamoto N, Mukae H, Fujii T, Kakugawa T, Kaida H, Kadota J, Kohno S (2004). Soluble form of Fas and Fas ligand in serum and bronchoalveolar lavage fluid of individuals infected with human T-lymphotropic virus type 1. Respiratory Medicine.

[ref-20] Sakamoto K, Taniguchi H, Kondoh Y, Wakai K, Kimura T, Kataoka K, Hashimoto N, Nishiyama O, Hasegawa Y (2012). Acute exacerbation of IPF following diagnostic bronchoalveolar lavage procedures. Respiratory Medicine.

[ref-21] Terashima T, Amakawa K, Matsumaru A, Van Eeden S, Hogg JC, Yamaguchi K (2001). BAL induces an increase in peripheral blood neutrophils and cytokine levels in healthy volunteers and patients with pneumonia. Chest.

[ref-22] Volpinari S, La Corte R, Bighi S, Ravenna F, Prandini N, Lo Monaco A, Trotta F (2011). Bronchoalveolar lavage in systemic sclerosis with lung involvement: role and correlations with functional, radiological and scintigraphic parameters. Rheumatology International.

